# A century of exercise physiology: key concepts in neural control of the circulation

**DOI:** 10.1007/s00421-024-05451-0

**Published:** 2024-03-05

**Authors:** J. Kevin Shoemaker, Robert Gros

**Affiliations:** 1 School of Kinesiology, The University of Western Ontario, London, ON N6A 3K7 Canada; 2 Department of Physiology and Pharmacology, The University of Western Ontario, London, ON N6A 3K7 Canada; 3 Department of Medicine, The University of Western Ontario, London, ON N6A 3K7 Canada

**Keywords:** Sympathetic nerve activity, Central command, Neurovascular transduction

## Abstract

Early in the twentieth century, Walter B. Cannon (1871–1945) introduced his overarching hypothesis of “*homeostasis”* (Cannon 1932)—the ability to sustain physiological values within a narrow range necessary for life during periods of stress. Physical exercise represents a stress in which motor, respiratory and cardiovascular systems must be integrated across a range of metabolic stress to match oxygen delivery to oxygen need at the cellular level, together with appropriate thermoregulatory control, blood pressure adjustments and energy provision. Of these, blood pressure regulation is a complex but controlled variable, being the function of cardiac output and vascular resistance (or conductance). Key in understanding blood pressure control during exercise is the coordinating role of the autonomic nervous system. A long history outlines the development of these concepts and how they are integrated within the exercise context. This review focuses on the renaissance observations and thinking generated in the first three decades of the twentieth century that opened the doorway to new concepts of inquiry in cardiovascular regulation during exercise. The concepts addressed here include the following: (1) exercise and blood pressure, (2) central command, (3) neurovascular transduction with emphasis on the sympathetic nerve activity and the vascular end organ response, and (4) tonic neurovascular integration.

## Overview

Landmark studies in the 1903–1932 period emerged along three disparate lines of inquiry and led to the current understanding of how the autonomic nervous system modifies the cardiovascular system in moments of exercise stress. These papers include Krogh and Lindhard’s (Krogh and Lindhard 1913) suggestion of a central cerebral cortex neural mechanism that coordinates cardiovascular control with motor function, a concept we now call central command. A second paper was published in 1932 by E.D. Adrian and his group (Adrian et al. 1932) that highlighted their work in direct electrical recordings of sympathetic postganglionic sympathetic nerves in mammals, providing a pathway to study signals with direct inference to events in the brain. Third, in 1933, W.B. Cannon first defined circulating catecholamines (Cannon 1933) as neurotransmitters linking sympathetic neurotransmission with vascular cellular responses (what we refer to as sympathetic neurovascular transduction) that had been introduced by Langley (1907, 1908) who was studying the concept of a cellular receptor that binds adrenergic chemicals (or drugs) to affect vascular cell function. The concept of neurovascular transduction has become a critical element in understanding muscle blood flow during exercise.

**Fig. 1:**
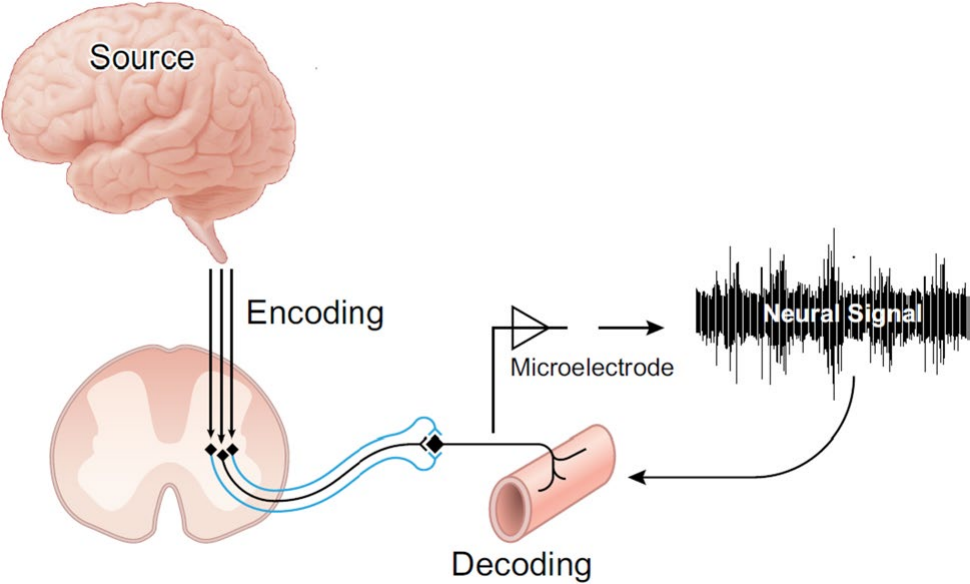
Major anatomical segments contributing to neural control of the circulation as depicted in discharge patterns in the postganglionic sympathetic neural signal. This figure expresses the ideas that central projections from the cortex merge with nuclei in the brainstem and, through variations discharge patterns affect a titrated regulation of vascular resistance. Missing from this figure are the vagal projections to the heart. Reproduced from (Shoemaker et al. 2018)

These landmark studies, emerging along disparate pathways, were establishing the framework to understand how the autonomic nervous system interacts with the cardiovascular system to form a highly integrated system that enables cardiovascular adjustments and adaptations to the stress of physical exercise. Figure 1 illustrates the integrated and connected nature of the brain, autonomic nervous system and cardiac or vascular end organs that are captured in the overall concept of Neural Control of the Circulation.

### Concept 1: exercise and blood pressure

**Fig. 2:**
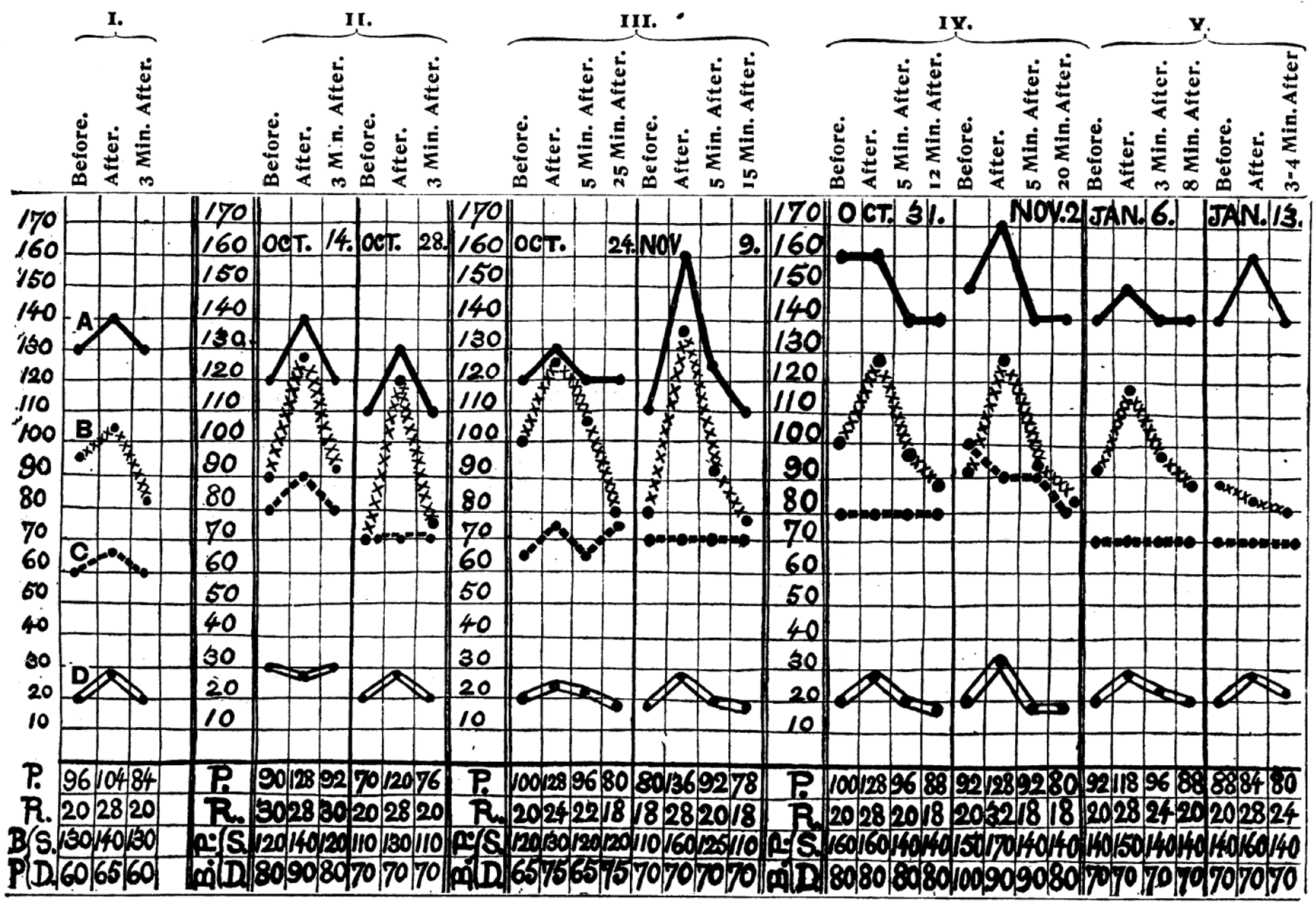
Systolic (**A**), diastolic (**B**) and mean (**C**) blood pressure together with ventilation rate (**D**) before, during and after exercise. Raw values are provided in the bottom four rows for pulse interval (Pi), respiration rate (R), systolic (B) and diastolic (P) pressure. From (Lambert 1918) with permission

**Fig. 3:**
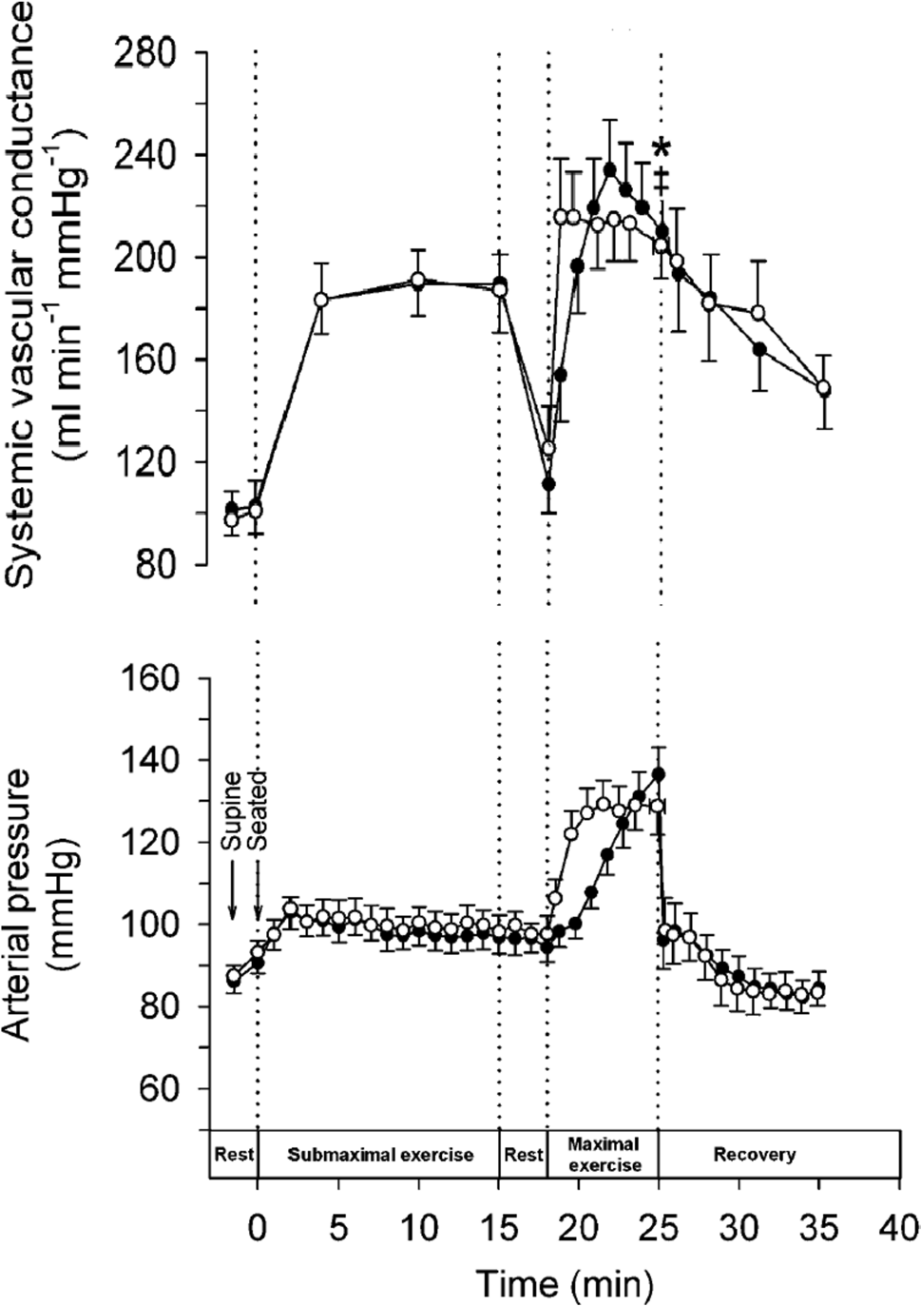
**S**ystemic vascular conductance and arterial blood pressure at rest, during submaximal and maximal exercise and 10 min of recovery performed during incremental (***•***) and constant load (○) exercise. Notice the plateau and reversal of vascular conductance during final minutes of maximal exercise. Data are means ± S.E.M. for 7–8 subjects, ∗ Lower than the value after 22 min when cycling at 80% of peak power, *P* < 0.05. ^***‡***^Lower than the peak values observed after 20–24 min of constant load maximal cycling, *P* < 0.05. From (Mortensen et al. 2005) with permission

**Fig. 4:**
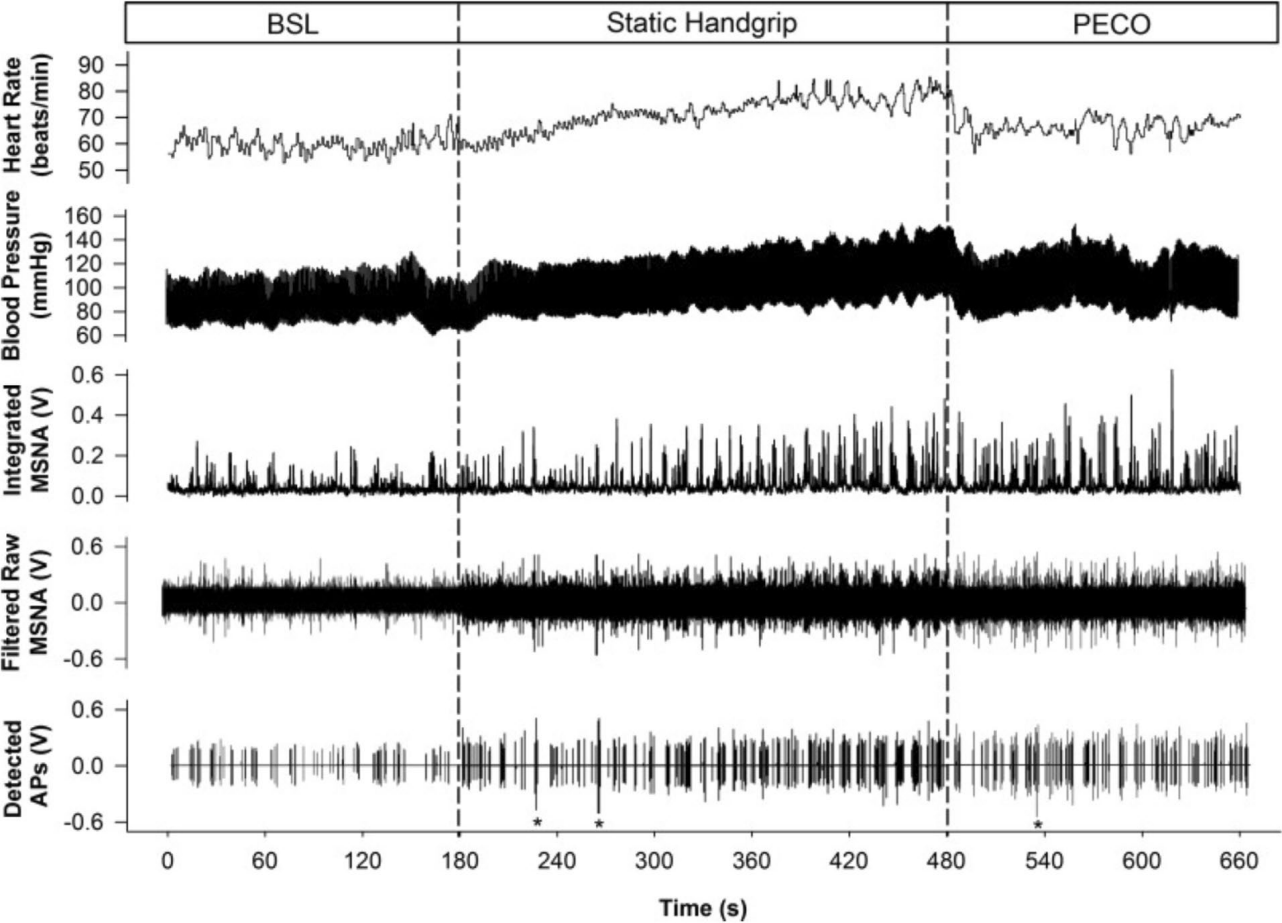
Representative data from a single subject illustrating the cardiac, hemodynamic and sympathetic responses at baseline (BSL), during static handgrip exercise and a period of postexercise circulatory occlusion (PECO).. HR, heart rate; BP, blood pressure; MSNA, muscle sympathetic nerve activity; AP, action potential. *Denotes noise spikes not included in analysis. From (Badrov et al. 2016b) with permission

The reporting of changes in blood pressure during exercise emerged in the 1880s (see (Otis 1911) for review of the original literature). One of the first reported recordings of blood pressure as a function of prolonged exercise was reported in 1907 (Gordon 1907). These early studies provided the consistent observation that the BP response to long-term submaximal whole-body exercise is small (e.g. ~ 10 mmHg). Figure 2 presents an example of the earliest data on blood pressure responses to whole-body dynamic exercise. However, in contrast to moderate intensity exercise, blood pressure increases markedly by ~ 50 mmHg during the latter moments of incremental whole-body exercise (Mortensen et al. 2005) (Fig. 3, lower panel) or sustained isometric contractions (Mitchell 1990), shown for a single individual in Fig. 4. Notable in Fig. 4 is the role of volitional effort to sustain the heart rate responses to exercise whereas peripheral sensors in fatigued muscle are critical for the sympathetic response, as indicated by the persistence of elevated sympathetic nerve activity during a period of post-exercise circulatory occlusion when volitional effort is stopped but the reflex drive continues.

This concept of the exercise pressor response to fatiguing exercise is firmly entrenched in our current understanding of exercise physiology. The mechanisms mediating this response, however, are multifactorial and have been debated, addressing important questions regarding the role of the heart versus vasoconstriction in the blood pressure increase as well as the role of reflex-mediated effects (Kaufman and Forster 1996; Kaufman et al. 1983) that interact with central perceptual features of “effortful” work (Mitchell et al. 1983). Of these factors, the potential role of cardiac function in exercise blood pressure regulation is often overlooked. For example, because efferent sympathetic nerve activity increases concurrently with blood pressure during intense exercise, it has been assumed that peripheral vasoconstriction largely accounts for the increased blood pressure. However, during fatiguing isometric handgrip exercise in human models (Shoemaker et al. 2000; Shoemaker et al. 2007), sympathetic nerve activity rises but cardiac output accounts for the bulk of the Ohmic rise in blood pressure rather than systemic vascular resistance, a conclusion also reported in a dog model of flow-restricted exercise (O'Leary and Woodbury 1996). In contrast to isometric models, blood pressure regulation during dynamic or whole-body exercise is more complex. For example, sympathetic nerve activity, as measured by microneurography techniques, may decrease during the early moments of moderate intensity cycling or knee extension exercise, and then increase markedly at exercise intensities above 40–50% of maximal strength (Ichinose et al. 2008; Katayama and Saito 2019). These patterns align with a reduced rate of increase (or even reversal) in vascular conductance as one approaches maximal workload (Mortensen et al. 2005) (see Fig. 3, upper panel).

Regulation of cardiac output during exercise involves, in part, a specific role for regional variations in alpha-adrenergic sympathetic vascular and venular constriction in the gut that reduce vascular capacitance in this region (Rowell 1986) so that more blood is directed back to the heart to support total cardiac output. However, the role of sympathetic activation towards both active (Boulton et al. 2021) and quiescent (Shoemaker et al. 2000) skeletal muscle, remains poorly understood, particularly when the leg vascular response can be accounted for by a myogenic constrictor effect secondary to the rise in blood pressure (Shoemaker et al. 2000). In addition, the larger release of epinephrine, together with heightened sympathetic nerve activity, observed during fatiguing exercise compared to other sympathetic stressors (Dyson et al. 2006) suggests that the balance between beta adrenergic receptor-induced dilation and alpha-adrenergic vasoconstriction may be altered in favour of dilation.

An additional and incompletely understood question regarding the exercise pressor response relates to the underlying peripheral and central neural mechanisms. Sensory receptors in skeletal muscle that detect altered metabolism and tension contribute reflex-mediated cardiovascular adjustments (Kaufman et al. 1983). However, the additional observations that heart rate and blood pressure increase together has led to the concept that the baroreflex set point can shift, essentially “requesting” a rise in blood pressure. A detailed exploration of this concept has been provided earlier (Raven et al. 2002). Mechanistically, direct electrophysiological and experimental approaches in anesthetized or decerebrate models indicate that descending neural inputs from the mesencephalic locomotor region converge with ascending thin muscle afferents (Type III afferents) and baroreceptor afferents in the nucleus tractus solitarius that modify baroreflex sensitivity (Degtyarenko and Kaufman 2006; McIlveen et al. 2001; Potts 2002). In humans, functional neuroimaging data point to involvement of higher cortical centres such as the medial prefrontal cortex and insula cortex (Williamson 2015) (see Central Command section below).

### Concept 2: central command

**Fig. 5:**
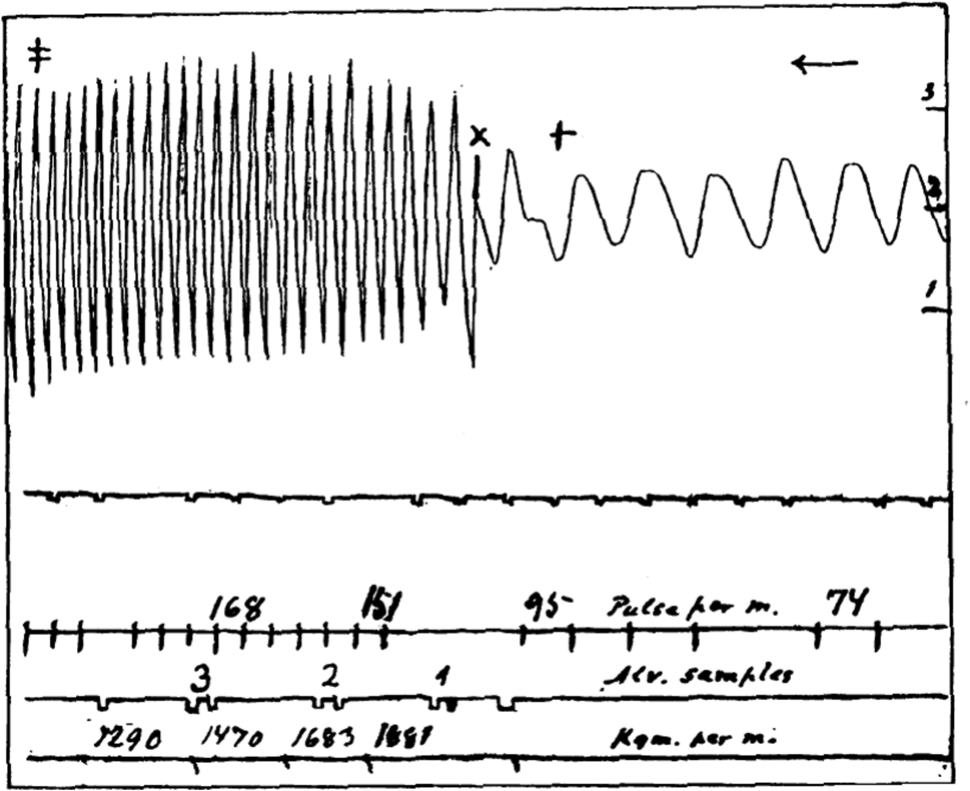
Original Fig. 1 from (Krogh and Lindhard 1913) illustrating the early rise in heart rate (pulse per minute) and ventilation during the transition from rest to moderate intensity cycling exercise. Arrow, the tracings flow from right to left in time. Time in 1/10 min; + , “ready”; X, “begin”; ≠ , “stop”. Ventilation scale (right ordinate) is litres/min. Reproduced with permission from the Journal of Physiology

In 1913, Krogh and Lindhard (1913) observed in a small sample of healthy individuals a rapid respiratory and tachycardia response immediately at the onset of, and sometimes in anticipation of, whole-body exercise, particularly heavy exercise. Figure 5 provides the original observation. Having ruled out options for a metabolic cause, they interpreted the rapidity of the responses to be caused by neural factors. They proposed this neural mechanism “irradiated” from the motor cortex to include respiratory and cardiovascular control. This study led to a continuing search for brain actions and anatomy that coordinate neural control of the circulation with those that affect skeletal-motor and respiratory function. In 1971, the term “central command” was used to describe this neural pattern (Goodwin et al. 1972), a name that is now used routinely.

In addition to the observations made by Krogh and Lindhard (1913), accumulating observations point to an important role for cerebral cortex sites in the regulation of autonomic function. For example, Walter B. Cannon’s characterization of “voodoo death” in 1942 illustrated the important and sometimes fatal role that psychologic stress can have on cardiovascular function (Cannon 1932). Clinical observations made in the 1980’s indicated that fatalities following cerebral stroke often were due to catastrophic cardiac arrhythmias (Cheung and Hachinski 2003; Myers et al. 1982), particularly when that stroke involved the insula cortex due to its ability to influence cardiopathologic outcomes from hyperadrenergic activation (Yoon et al. 1997; Yasui et al. 1991; Oppenheimer et al. 1991). Additionally, studies on individuals with cortical lesions demonstrate the role of the medial prefrontal cortex in modulating cardiovascular function. In particular, these individuals show blunted emotion, poor decision-making and a failed alteration in the skin conductance response that is mediated mainly by the sympathetic nervous system (Bechara et al. 1996, 1997; Damasio 1994).

Defining the specific higher brain regions serving a role in central command for exercise-based cardiovascular arousal has proven to be a significant challenge with several barriers. First, a conscious animal preparation is required that can perform volitional work, with corresponding cortical activity unimpaired by anesthesia. Second, centrally mediated effects must be separated from rapid reflexive effects. Third, methods are required that expose functional outcomes in the cerebrum with sufficient spatial resolution to study specific candidate brain regions as well as temporal resolution to catch neural patterns as they occur, particularly if examining regulation of the rapid changes in heart rate at the exercise onset. Finally, peripheral metrics of autonomic function are required to fully interpret central neural influences.

Functional evidence exposes volitional effort as an important component of the central command. Using a conscious feline model Matuskawa’s group illustrated greater cardiac and blood pressure responses to active changes in posture compared to a passive lifting of the animal into the same position (Ishii et al. 2023). In humans, measures of muscle sympathetic nerve activity (MSNA- discussed in detail below in Concept 3) and the electrocardiogram indicate that heart rate responses to brief handgrip contractions are rapid and express a dose response relationship with workload whereas sympathetic responses are delayed during submaximal static exercise (Seals and Victor 1991). Otherwise, a strong muscular contraction (i.e., > 70% of maximal effort) is needed to generate an immediate sympathetic burst (Gandevia and Hobbs 1990; Mitchell and Victor 1996). Pharmacological blockade studies indicated that the rapid heart rate response at the onset of exercise is due to alterations in vagal dominance over cardiac function (Victor et al. 1987). Inasmuch as “effort” reflects a central command characteristic, these studies suggest that central pathways may affect cardiovagal control differently than sympathetic responses.

The search for sites of the brain that mediate the coordinated cardiovascular, respiratory and skeletal motor responses to exercise begins with a study of the general cortical pathways that are associated with, and/or affect, cardiovascular function. Primarily using anesthetized rat models, a series of studies over the past four decades have summated to a general knowledge of the cortical autonomic network in that species (Benarroch 1993; Cechetto and Saper 1990), emphasizing the hypothalamus, prefrontal cortex, insula cortex, amygdala, and hippocampus. The 1990s brought major breakthroughs in studying cerebral structures involved in cardiovascular control in conscious humans. The first was the development of blood oxygen level-dependent (BOLD) neuroimaging using magnetic resonance imaging (Ogawa et al. 1992). The second was surgical implantation of stereo-electroencephalographic depth electrodes that could be used to stimulate discreet brain regions (Oppenheimer et al. 1992). These techniques exposed the functional cortical anatomy that drives cardiovascular outcomes, and b) the cortical activation patterns that correlate with cardiovascular outcomes such as heart rate during exercise.

Nowak et al. (1999, 2005) investigated cortical outcomes when paraplegic subjects attempted a foot lift and reported activation in the cerebellum and insular cortex, with the insula being activated under conditions involving isolated feed forward control. Williamson et al. (2001) used hypnosis to change the perception of effort during a constant load cycling exercise. Contrasting the baseline exercise with the condition of perceived heavier workload (i.e. effort sense) they reported corresponding changes in perceived effort and cortical activation levels in the anterior cingulate cortex, insular cortex and thalamic regions. Notably, no differences were found in areas related to motor performance indicating that the observed brain activation patterns were not related to a change in work performed. Williamson et al. (2002) also utilized hypnosis to compare patterns of cortical activation between actual and imagined handgrip exercise. They reported activation of the insular and anterior cingulate cortex and these regions appeared to be involved in cardiovascular modulation independent of any muscle afferent feedback. While different sites are reported in different models, common patterns emerged from these early studies pointing to the insula cortex and cingulate cortex.

Recent developments in surgically implanted electroencephalography electrodes to explore sites related to refractory epileptic seizures have opened new avenues to study the cortical sites associated with cardiovascular control in conscious humans. While rare, studies are emerging to explore the impact of regions in the cortical autonomic network and their impact on autonomic function. For example, direct electrical stimulation models in both rodents and humans point to an important role for the insula cortex in cardiac function (Butcher and Cechetto 1995a, b; Cechetto et al. 1989; Oppenheimer and Cechetto 1990; Oppenheimer et al. 1991). However, caution is encouraged in interpreting the insula as a homogenous region of the brain in its function. For example, using electrical stimulation of over 100 sites in the IC of patients with epilepsy Chouchou (2019) reported that elevated activity (via electrical stimulation) of the anterior and median insula regions primarily caused *bradycardia* with posterior insula stimulation primarily causing tachycardia. In contrast, functional imaging results during volitional handgrip exercise that *elevates* HR indicate a role for anterior insula activation in exercise tachycardia (Shoemaker et al. 2015b). Also, in a case study, direct stimulation of the right inferior posterior insula was associated with bradycardia at baseline and an impaired heart rate response to handgrip exercise (Al-Otaibi et al. 2010) whereas stimulation of the superior posterior insula had no effect. Reconciling the conflicting data of similar anterior insula activation patterns but disparate HR responses outlined above remains difficult. The answer(s) may lie in the complex attributes of the insula such as its many subdivisions (Macey et al. 2012), its highly viscerotopic organization for sensory inputs from muscle, gut, and vagus nerve (i.e., cardiac inputs) (Cechetto 1987; Cechetto and Saper 1990), and its role in processing viscerosensory inputs with motor/behavioural outcomes. Additionally, the integrated contributions of IC may vary when engaged during volitional mechanisms that engage an entire network versus local stimulation of a single site within the network.

While electrical recordings from the brains of animals preceded functional neuroimaging models, the non-invasive allowances of the latter approach have enabled tremendous advances in the field. This approach repeatedly points to a group of regions that associate predictably in cardiovascular regulation (Shoemaker et al. 2012; Cechetto and Shoemaker 2009; Ruiz Vargas et al. 2016; Vargas et al. 2016; Beissner et al. 2013; Thayer et al. 2012; Henderson et al. 2012; Critchley et al. 2000) (Shoemaker 2022). These regions include the medial prefrontal cortex, the insula cortex, amygdala, hippocampus, dorsal anterior cingulate cortex, posterior cingulate cortex, dorsolateral frontal cortex and hippocampus. It is noted that some regions are exposed during autonomic challenges such as breath holds or exercise whereas others are apparent under baseline conditions with regional activation oscillations that correlate with cardiovagal function (Thayer et al. 2012) or efferent sympathetic nerve activity directed to skeletal muscle (Henderson et al. 2012).

Nonetheless, when used during volitional activity models, functional neuroimaging is not able to separate sensory from motor or inhibitory versus excitatory neural patterns. One example of complicating factors that might interfere with detection of discreet cortical neural functional patterns using muscle contractions is the sensory input to the brain from the muscle that will report the extent of muscle tension through muscle spindle afferents, or metabolic stress through muscle chemo/metaboreceptors. We have studied the potential impact of muscle spinal somatosensory afferents in patterns of brain activation in contrast to those achieved during involuntary and voluntary muscle activation (Goswami et al. 2011). These studies indicated that whereas volitional muscle activation produced reduced activation in the medial prefrontal cortex and increased anterior insula activation (among other patterns), transcutaneous electrical stimulation of somatosensory afferents led to the opposite patterns (decreased anterior insula activation and elevated medial prefrontal cortex activity). Therefore, in models of conscious volitional work, such sensory inputs could rapidly modulate cortical pathways associated with cardiovascular adjustments to motor activity, potentially mitigating opportunities to observe regions related to central command independent of sensory inputs. Studies incorporating disruptions of sensory inputs during exercise are not reported to our knowledge.

The statement made by Jon Williamson (2015) outlines the current state of our knowledge regarding central determinants of autonomic cardiovascular control in exercise: “Several studies have attempted to address these issues and provide more definitive neuroanatomical information. However, none have clearly answered the question, “where is central command?”. Difficulties in peering into the brain of a conscious human, complexities of neural networks, and redundant pathways under feedback regulation continue to represent fundamental challenges to studying cortical autonomic function. Important reviews of the current state of knowledge regarding Central Command include (Williamson 2015; Padley et al. 2007).

### Concept 3: neurovascular transduction

#### Sympathetic nerve activity to skeletal muscle

In the 1920s and 1930s, Adrian was studying the general discharge properties of nerves and produced a foundational document in 1932 (Adrian et al. 1932) providing the first recordings postganglionic sympathetic nerves in mammals. A critical element of this discovery lies in the provision of a method that gains direct access to centrally mediated sympathetic neural information. These recordings highlighted the fundamental properties of postganglionic sympathetic nerve activity that include a neurogram with poor signal-to-noise and a characteristic rhythmic neuronal activity that normally is entrained to the cardiac cycle, respiratory patterns and to blood pressure oscillations. Further study of neural rhythms can reveal how the sympathetic nervous system is wired with other complex networks. For example, searches for the cause of the respiratory rhythms exposed different types of C1 and non-C1 populations of pre-sympathetic neurons in the rostral ventrolateral medulla that, through impacts of neurons from pre-Botzinger neurons that drive respiratory patterns, affect rhythmic features of efferent post-ganglionic activity (Moraes et al. 2013; Menuet et al. 2020).

##### Microneurographic measures of postganglionic sympathetic nerve activity in conscious humans

Direct measures of sympathetic nerve activity directed to skeletal muscle vasculature (MSNA) were first made in humans by Hagbarth and Vallbo (1968). A detailed review of the development of the microneurographic technique has been provided recently (Vallbo 2018). This work represented a major leap forward in autonomic neuroscience by enabling access to, and routine measures of, the efferent sympathetic neural signal in superficial nerves of conscious humans with signals that faithfully represent preganglionic discharge patterns (see (Shoemaker et al. 2018) for review). In addition to conscious state measures, this breakthrough opened the door for measures of sympathetic nerve activity while participants experienced or performed varying stress-inducing tasks including exercise (Delius et al. 1972; Sundlof and Wallin 1978; Vallbo et al. 1979; Wallin et al. 2003), enabling tremendous growth in our knowledge of efferent sympathetic nerve activity in basic studies of reflex cardiovascular control, as well as clinical studies into the impact of primary and secondary dysregulation of the sympathetic nervous system on human disability.

Previous reviews highlight the changes in MSNA in response to small muscle-mass isometric contractions (Seals and Victor 1991) and dynamic exercise (Katayama and Saito 2019). In brief, the pattern and magnitude of MSNA responses to exercise depend on factors such as mode, intensity and duration of the exercise. In either isometric or dynamic models, the MSNA response appears to be coupled with the progression of muscle fatigue; however, some variations occur. In moderate intensity isometric models, there is little change in MSNA at the exercise onset (Seals and Victor 1991) lasting about 30 s for a moderate intensity contraction. It can be surmised that the subsequent increase in MSNA that occurs with the sustained contraction is linked to muscle metabolic reflex inputs because the sympathetic activation continues during a post-exercise period of limb ischemia that traps metabolites produced during exercise (see Fig. 4). In dynamic contractions, particularly those involving larger muscle mass, a transient inhibition of MSNA can be observed at the exercise onset (Katayama and Saito 2019): this inhibition appears to be related to cardiopulmonary baroreceptor inhibition of MSNA due to the pumping action of contracting muscle to shift blood back to the heart. Thereafter, MSNA levels increase markedly with the onset of fatigue signalling the influence of both muscle reflexes as well as a role for central “effort” as illustrated by the post-exercise circulatory occlusion approach mentioned above.

One of the rhythms found in efferent muscle sympathetic nerve activity reflects synchronization of efferent axonal activity in bursts that are entrained to the cardiac cycle through baroreflex neural pathways. The bursts vary in their frequency and size. These patterns are observed under resting conditions and during stress such as incremental exercise where both burst frequency and burst size increase. The bursty pattern appears to be critical to the end organ response (Ninomiya et al. 1993; Kluess et al. 2006), providing a greater vascular outcome than constant sympathetic outflow. Furthermore, when measured during baseline conditions, larger bursts elicit a larger reduction in vascular conductance (Fairfax et al. 2013b) that is sensitive to alpha adrenergic receptor blockade (Fairfax et al. 2013a) indicating the role of norepinephrine released from these neurons. Therefore, sympathetic nerve rhythms not only reflect wiring of the reflex and central networks, but also a unique physiological relevance to the end organ response.

In addition to physiologically relevant rhythms, the MSNA bursts exhibit other unique features. For example, Wallin et al. (1994) first reported the timing of sympathetic bursts relative to the baroreflex-mediated termination of a burst. Specifically, under conditions of physical rest, larger bursts seem to travel faster along the brainstem-to-fibular nerve or radial nerve recording sites, causing them to propose the ideas that either a subpopulation of fast-conducting (i.e., larger diameter) axons exist that are not always active, or the presence of unique and modifiable synaptic delays affected burst timing. Concurrently with Wallin’s provocative report (Wallin et al. 1994), additional groundwork for this hypothesis was provided by Macefield and Wallin (1994) who adapted the microneurography technique with higher impedance electrodes to emphasize the activity of single sympathetic efferent axons that discharged primarily with a probability of 1 Hz (once/burst) but with variable latencies. To see if subpopulations of latent neurons existed, being recruitable either spontaneously or during reflexive stimulation (Marshall et al. 1961), and following from the preliminary work performed by Diedrich et al. (2003), a wavelet-based detection method was developed to isolate and study all axons in the multi-unit sympathetic neurogram as measured with lower impedance electrodes that provided a wider recording field (Salmanpour et al. 2010). The series of studies that emerged exposed variously sized axons (Tompkins et al. 2013) with varying firing probabilities whereby medium sized axons provided the highest probability of firing and are under the greatest control by the baroreflex (Klassen et al. 2020; Klassen and Shoemaker 2021). Under baseline conditions, the disappearance of MSNA action potentials following ganglionic blockade progresses from largest to smallest (Klassen et al. 2018). Some larger axonal action potentials not present at baseline become evident only during severe chemoreflex (Steinback et al. 2010) or exercise (Badrov et al. 2016a) stress. These features are interesting in that they expose a hierarchical recruitment strategy within the sympathetic nervous system, as well as a neural system that appears to use probability coding, population coding and temporal coding strategies to convey information to the end organ.

The sympathetic discharge patterns mentioned above, particularly those of recruitment of latent subpopulations during severe stress, create at least two follow-up questions: (1) what central mechanisms determine these patterns, and (2) what is(are) the functional purpose of these recruitment patterns? Since measures of postganglionic sympathetic nerve activity provide direct access to signals arising from the brainstem where integration of top-down cortical influence and visceral reflex sensory information occurs, the discussion of Central Command outlined above likely provides some insight into cortical sites that might have some impact on these features. Several recent observations provide support for this idea. Correlations were found between cortical thickness in regions of the cortical autonomic network and sympathetic nerve activity as well as heart rate variability indices of cardiovagal function (Wood et al. 2017). Further, neuroimaging studies highlight correlations between brain regions within the cortical autonomic network and fibular nerve recordings of baseline sympathetic nerve activity (Fatouleh et al. 2014; Henderson et al. 2012). Importantly, recruitment of latent sympathetic axonal subpopulations occurs during fatiguing exercise but not during post-exercise circulatory occlusion (Badrov et al. 2016a) indicate that central mechanisms underly latent axonal recruitment in this context. Also, arousal during sleep elicits K-complexes in electroencephalographic scalp recordings that correlate to the size and latency of bursts in muscle sympathetic nerve activity (Xie et al. 1999). These observations support the hypothesis that the cerebral cortex influences efferent sympathetic discharge patterning.

Why do variations in sympathetic recruitment and action potential firing probabilities exist? Currently, this question has not been addressed experimentally. We do know that the size of each integrated sympathetic burst depends on the size and number of action potentials that are synchronized in that cardiac cycle (Salmanpour et al. 2011; Steinback et al. 2010), that during stress (including exercise (Badrov et al. 2016a)) larger action potentials become apparent indicative of recruitment of latent subpopulations, and that larger bursts elicit a larger vasoconstrictor outcome (Fairfax et al. 2013a; Fairfax et al. 2013b). These observations suggest that the variable and hierarchical recruitment patterns enable a fine-tuning of total efferent sympathetic outflow as well as vasoactive control. These variations in sympathetic recruitment and firing probabilities may affect variations in neurotransmitter release from sympathetic nerves. These patterns also provide a possible mechanism to support the co-transmission of different vasoactive neurotransmitters, as reported by Geoffry Burnstock (1985). This topic has been reviewed previously (Shoemaker et al. 2015a).

##### Catecholamines

Focusing primarily on baseline conditions in animal or in situ preparations, a major linkage between recordings of sympathetic nerves by Adrian (1932) and vasoconstriction was provided by Cannon who first defined circulating catecholamines in 1933 (Cannon 1933) as neurotransmitters. Concurrently, the concept of vascular cellular responses to catecholamines was introduced by John Newport Langley (1907, 1908) who was studying the idea of a cellular receptor that binds chemicals (or drugs) to affect cell function. It is understood now that noradrenaline has a neural source whereas epinephrine primarily is produced in the adrenal glands in response to noradrenergic activation, although spill-over measures indicate some epinephrine may have a neural source too (Esler et al. 1991). The measures of organ-specific norepinephrine release in the 1980s (Esler et al. 1988), the influence of sympathetic discharge pattern on vascular constriction (Fairfax et al. 2013a), release of purines (Burnstock 1999), adrenergic and peptide-based sympathetic neurotransmitters (Pernow et al. 1989), and the concept of sympathetic co-transmitters from the same axon (Pablo Huidobro-Toro and Verónica Donoso 2004; Burnstock 1995) have begun to fill the gaps in understanding the complexity of transduction of sympathetic nerve activity into a constrictor response in the vascular end organ.

As introduced above, a potentially critical aspect regulating neurotransmitter release is the neural discharge patterns. For example, Kluess et al. (2006) reported that P2X receptors and α1-receptors in the femoral artery are sensitive to frequency and patterns of electrical stimulation of the sympathetic nerves and are decreased in continuous versus “bursty” patterns of stimulation. Furthermore, by assessing patterns of intracellular calcium transients, Wier et al. (2009) demonstrated variations in the mechanisms, timing and magnitude of constrictor effects elicited by P2X, alpha adrenergic and Y1 receptor activation. However, how patterns of recruitment in efferent sympathetic post-ganglionic axonal subpopulations associated with exercise affect release of purines, norepinephrine and/or neuropeptide Y, and how these patterns are regulated either directly or indirectly by supramedullary brain sites or neural pathway modulators between the brain and vascular end organ, remain unknown.

### Concept 4: Tonic sympathetic vasoconstriction in active skeletal muscle

Exercise blood pressure regulation depends on the sympathetic nervous system’s ability to titrate the relationship between systemic vascular conductance on one hand, and, on the other, the heart’s ability to support that conductance through the volume of cardiac output. Total blood flow capacity of contracting skeletal muscle is very high, being linearly related to the workload. Specifically, Andersen and Saltin (1985) estimated that leg blood flow during maximal effort cycling exercise could reach 250 ml/min/100 gm of tissue. During whole body exercise this magnitude of blood flow would outstrip available cardiac output raising the question of why hypotension does not occur during heavy exercise performed by healthy intact individuals. Of note, the high leg blood flow observed by Saltin’s group during single-legged dynamic exercise was diminished when the exercise was performed by two legs (Magnusson et al. 1984) indicating a constraint on total flow capacity that was affected by the mass of muscle activated. Integrative analysis of these features provided by Niels Secher et al. (Mortensen et al. 2005) illustrated in elite athletes an increase in systemic vascular conductance with exercise until cardiac output was maximal at which time the work rate-induced rise in systemic vascular conductance was stopped and even reversed, observations that are supported by more recent reports in humans (Volianitis and Secher 1985; Travers et al. 2022) and previously in dogs (O'Leary et al. 1997b). Moreover, systemic blockade of sympathetic outflow in dogs resulted in syncope as the treadmill work rate increased (Sheriff et al. 1993). Additionally, activation of the carotid baroreflex to reduce sympathetic outflow during exercise (Vatner et al. 1970; Collins et al. 2001) revealed substantial vasoconstriction in active skeletal muscle. Finally, studies of inadequate sympathetic vasomotor control can provide further insight into the critical role sympathetic nerve activity in exercise tolerance. Key studies in this regard were published in the early 1960s such as Marshall et al. (1961) who measured a 30–50 mmHg decrease in arterial blood pressure in six individuals diagnosed with idiopathic hypotension during exercise regardless of whether this exercise was performed in the supine or upright postures and, thereby, regardless of variations in orthostatic venous pooling. This group suggested the exercise-induced hypotension, as measured in these patients, was due to the failure of compensatory constriction of the vascular beds and not of cardiac output. Therefore, effective sympathetic vasomotor control represents a critical element of the integrated responses to exercise that regulate blood pressure and blood flow. The major hypothesis here (Rowell 1993; Rowell et al. 1996) is that dilated muscle during maximal exercise represents a key target organ for the sympathetic nervous system to regulate blood pressure whereby constraint of that dilatory response, coupled with a high cardiac output, achieves the blood pressure necessary to perfuse the active muscle.

A role for sympathetic vasoconstriction also exists under baseline conditions. Experiments performed by Claude Bernard (1813–1878 AD) and Brown-Seguard (1817–1894 AD) on the impact of severed nerves on blood flow provided the first evidence of active neurogenic control over cardiovascular function under baseline conditions (as cited in Cooper 2008 and Bing et al. 1982). Similar conclusions were made much later, illustrating the direct vascular impact of sympathetic adrenergic receptor blockade (O'Leary et al. 1997a), sympathetic nerve release of norepinephrine (Shoemaker et al. 1999), and anesthetic blockade of sympathetic nerve fibres (Joyner et al. 1992). This concept of tonic sympathetic vasoconstriction at rest and during exercise is now part of our fundamental knowledge regarding cardiovascular control.

## Summary

**Fig. 6:**
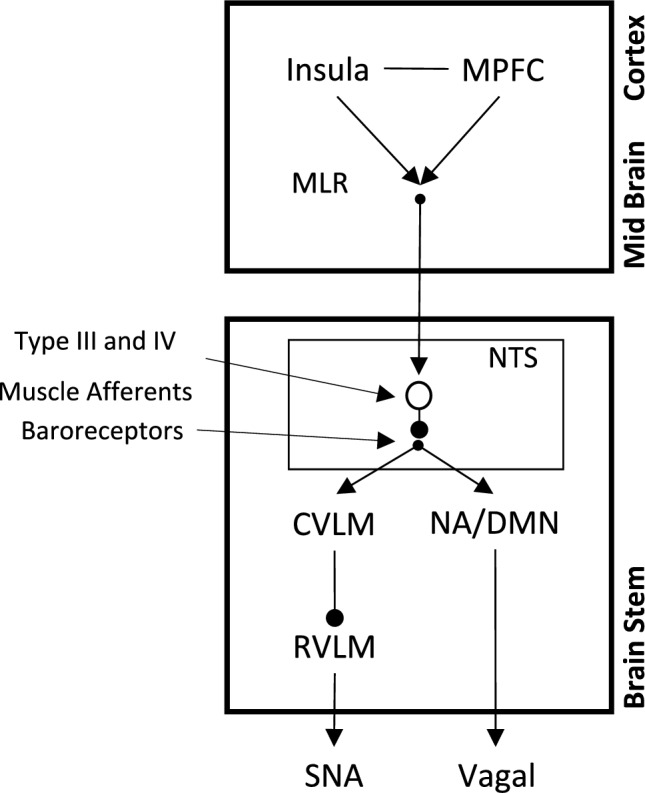
Schematic representing probably pathways that integrate central command, baroreflex resetting, muscle activation and autonomic adjustments to exercise. Top-down signals related to volitional effort or action planning (involving Insula, medial prefrontal cortex (MPFC) and/or mesencephalic locomotor region (MLR)) activate pathways in the nucleus tractus solitarius (NTS) to affect sympathetic nerve activity (SNA) and cardio-vagal control. This pathway is affected by sensory inputs from baroreceptors and skeletal muscle afferents to affect sympathetic nerve activity (SNA) and vagal outflow. *NTS*, nucleus tractus solitarius; *CVLM* caudal ventrolateral medulla; *RVLM*, rostral ventrolateral medulla; *NA*, nucleus ambiguous; *DMN*, dorsal motor nucleus)

Landmark studies in the 1903–1932 period along three disparate lines of inquiry opened investigations that have contributed significantly to the current understanding of how the autonomic nervous system modifies the cardiovascular system during exercise. These papers include Krogh and Lindhard’s (1913) suggestion of a central cerebral cortex neural mechanism that coordinates cardiovascular control with motor function, a concept we now call central command. While our knowledge regarding central contributions to autonomic function remain incomplete, studies using different models suggest that neural linkages from cortical and midbrain regions, including the insula cortex, prefrontal cortex, hippocampus and mesencephalic locomotor regions of the midbrain, converge on baroreceptor sensory inputs to the nucleus tractus solitarius to affect a resetting of the baroreflex set point, allowing both blood pressure and heart rate to increase during exercise. Sensory inputs from muscle also impact these autonomic cortical sites to augment sympathetic drive and reduce vagal outflow, in the case of the Type III and IV muscle afferents. A summary of these integrated neural effects is presented in Fig. 6.

The ability to measure directly efferent sympathetic nerve activity in mammals was introduced in 1932 by Adrian and his group (Adrian et al. 1932). Following the development of microneurographic techniques that can be used in humans, the nature of the efferent sympathetic nerve activity has been explored in detail resulting in an understanding of action potential emissions that are synchronized by the baroreflex to produce bursts of activity that increase in size and frequency during exercise. While heart rate responses occur at the exercise onset, MSNA responses are delayed being modulated by cardiopulmonary baroreceptor loading during dynamic exercise and/or the requirement for important metabolic changes to occur as muscle work and fatigue ensure, as observed during static moderate intensity exercise. Nonetheless, marked increases in MSNA occur with incremental exercise and, when cardiac output is limited, exert a growing vasoconstrictor influence on vasculature in quiescent and active muscle, competing with the dilatory influences associated with engagement of skeletal muscle. The MSNA burst size and timing are important contributors to the vascular vasoconstrictor response.

Third, the concept of sympathetic neurotransmitters and vascular cellular responses (what we refer to as sympathetic neurovascular transduction) was introduced by John Newport Langley (1907, 1908) who was studying the concept of a cellular receptor that binds sympathetic chemicals (or drugs) to affect cell function and W.B. Cannon who first defined circulating catecholamines in 1933 (Cannon 1933) as neurotransmitters. The presence of co-transmitters ATP, NE and NPY is now recognized and their independent roles and mechanisms of action are being elucidated, although their effects in human models require additional study.

When combined, these landmark studies established the foundation upon which we have begun to understand how the autonomic nervous system interacts with the cardiovascular system to enable physiological adaptation to the stress of physical exercise.
